# Validation of Intrinsic Left Ventricular Assist Device Data Tracking Algorithm for Early Recognition of Centrifugal Flow Pump Thrombosis

**DOI:** 10.3390/life12040563

**Published:** 2022-04-09

**Authors:** Christoph Gross, Kamen Dimitrov, Julia Riebandt, Dominik Wiedemann, Günther Laufer, Heinrich Schima, Francesco Moscato, Michael C. Brown, Abhijit Kadrolkar, Robert W. Stadler, Daniel Zimpfer, Thomas Schlöglhofer

**Affiliations:** 1 Department of Cardiac Surgery, Medical University of Vienna, 1090 Vienna, Austria; christoph.gross@meduniwien.ac.at (C.G.); kamen.dimitrov@meduniwien.ac.at (K.D.); julia.riebandt@meduniwien.ac.at (J.R.); dominik.wiedemann@meduniwien.ac.at (D.W.); guenther.laufer@meduniwien.ac.at (G.L.); heinrich.schima@meduniwien.ac.at (H.S.); daniel.zimpfer@meduniwien.ac.at (D.Z.); 2 Ludwig Boltzmann Institute for Cardiovascular Research, 1020 Vienna, Austria; francesco.moscato@meduniwien.ac.at; 3 Center for Medical Physics and Biomedical Engineering, Medical University of Vienna, 1090 Vienna, Austria; 4 Austrian Cluster for Tissue Regeneration, 1200 Vienna, Austria; 5 Medtronic Inc., Minneapolis, MN 55432, USA; michael.c.brown1@gmail.com (M.C.B.); abhijit.kadrolkar@medtronic.com (A.K.); robert.stadler@medtronic.com (R.W.S.)

**Keywords:** left ventricular assist devices, mechanical circulatory support, patient monitoring, pump thrombosis

## Abstract

Advanced stage heart failure patients can benefit from the unloading effects of an implantable left ventricular assist device. Despite best clinical practice, LVADs are associated with adverse events, such as pump thrombosis (PT). An adaptive algorithm alerting when an individual’s appropriate levels in pump power uptake are exceeded, such as in the case of PT, can improve therapy of patients implanted with a centrifugal LVAD. We retrospectively studied 75 patients implanted with a centrifugal LVAD in a single center. A previously optimized adaptive pump power-tracking algorithm was compared to clinical best practice and clinically available constant threshold algorithms. Algorithm performances were analyzed in a PT group (*n* = 16 patients with 30 PT events) and a thoroughly selected control group (*n* = 59 patients, 34.7 patient years of LVAD data). Comparison of the adaptive power-tracking algorithm with the best performing constant threshold algorithm resulted in sensitivity of 83.3% vs. 86.7% and specificity of 98.9% vs. 95.3%, respectively. The power-tracking algorithm produced one false positive detection every 11.6 patient years and early warnings with a median of 3.6 days prior to PT diagnosis. In conclusion, a retrospective single-center validation study with real-world patient data demonstrated advantageous application of a power-tracking algorithm into LVAD systems and clinical practice.

## 1. Introduction

Left ventricular assist devices (LVADs) are an established therapy for advanced heart failure patients with reduced ejection fraction with an estimated population of 300,000 potential patients [1]. Incremental improvements in survival on LVAD and in long-term use of LVADs have opened up focus towards key research fields such as minimizing adverse events (AEs) and improving patient management and quality of life [2]. Major adverse events typically observed in LVAD patients include stroke and transient ischemic attack (neurological dysfunction), bleeding, infection, right heart failure, arrhythmias and device malfunction. These AEs are associated with frequent readmissions, lower survival and higher treatment costs [2].

Device malfunctions include device thrombosis and failure of LVAD electrical components. Device thrombosis, together with life-threatening device malfunction, can lead to embolic strokes. To prevent thrombi and embolic strokes, a target range INR of 2.0–3.0 [3] and blood pressure management with a mean arterial blood pressure below 90 mmHg are recommended [4]. Despite antithrombotic treatment, thromboembolic events cannot be prevented in all LVAD patients.

Freedom from first device malfunction and pump thrombus (PT) have been reported with 87.1% and 91.9% for 1 year after device implant and 54.2% and 72.8% after 5 years, respectively [5]. According to Molina et al. [5], the analysis of the Intermacs registry showed that 14.8% of patients with device malfunction and 8.8% of patients with PT corresponded to 0.16 device malfunctions and 0.09 PT events per patient year.

Thrombus formation can aggregate around the inflow cannula in the left ventricle (pre-PT), inside the pump, most likely on the impeller (intra-PT) or in the outflow graft (para-conduit device thrombus) [6,7,8]. Whereas all of these forms are generally referred to as device thrombus, the focus of the current study is on intra-PT. According to the updated definitions of AEs from the mechanical circulatory support academic research consortium, PT is classified as suspected or confirmed on the basis of biochemical, clinical and hemodynamic findings, based on incontrovertible imaging evidence or device inspection following device explant [2].

Intrinsic LVAD data monitoring or the controller’s alerting system can be advantageous with the communication of abnormal pump parameters, which may lead to the patient visiting the hospital for check-up and laboratory tests of blood drawn samples. These abnormal intrinsic pump data might be recognized on the pump controller’s display by the patient, a caregiver or a VAD clinician and interpreted subjectively. Different appearances in the pump power consumption trends have been reported, which can indicate pump thrombosis with a lead time of a few days or even containing information regarding the site of thrombosis formation [6,7,9,10].

LVAD logfile data can contain information on the patient’s hemodynamic status, cardiac parameters and pump–heart interactions. These time series data are highly patient-specific and sensitive to diurnal (circadian) variations, physical activity, LVAD speed settings and other factors [11]. Current alarms of clinically used LVADs are typically parameter threshold comparisons, such as the manually set HVAD (Medtronic Inc., Minneapolis, MN, USA) “high watt” alarm threshold, which is 2 W above the patient’s baseline power consumption [12]. This demonstrates a mismatch between simple alarms and complex physiologic data of the assisted cardiac and peripheral system. Consequently, thresholds are often set at a level to avoid or minimize alarms without “explainable reasons” (false positive). Therefore, a novel power-tracking algorithm has recently been developed for detecting abnormal changes in pump power that may be associated with PT using logfile data, with the potential for early PT warning [13]. Patient management tailored to the individual to avoid the risk factors associated with PT, combined with an alerting system using the power-tracking algorithm, could help to mitigate or even prevent the associated adverse effects, and ultimately improve LVAD patient outcomes.

The primary outcome of this study was to validate the performance of this self-adaptive algorithm for detecting abnormal pump performance parameters indicative of PT using retrospective real-world clinical data from patients undergoing HVAD implantation, including data on PT events and non-events. Secondary outcomes included comparison of the algorithm with constant power threshold for PT detection, stratified by observable power abnormalities classified by expert annotation, as well as the identification of demographic, hemodynamic and laboratory values as risk factors for PT.

## 2. Materials and Methods

In this retrospective, single-center study approved by the Institutional Review Board of the Medical University of Vienna (EK-Nr. 1769/2018, waiver of informed consent), logfile data from the LVAD device, blood laboratory tests, hemodynamics and demographics were collected from adult patients implanted with an HVAD.

### 2.1. Pump Logfile Data

HVAD logfiles contain pump performance log data including the parameters such as pump speed, power consumption, current and estimated LVAD flow, stored on the controller once every 15 min. Logfiles were downloaded and stored routinely when the patients were in the hospital for their medical checkup or due to readmission. Regular outpatient follow-ups are scheduled every 2 to 3 months [14]. Additionally, logfiles were received from a different hospital due to readmission, from a patient or a caregiver after calling the hospital’s out-patient LVAD hotline or during the periodically performed telephone check-ups [15]. Pump controller logfiles were converted into one aggregated pump data log for each patient and data point duplicates were deleted. Expected power ranges according to the pump’s set speeds were used for normalization of pump power trends, calculated with the formulas reported by Chorpenning et al. [16].

### 2.2. Patient Cohorts

The pump thrombus group consisted of the patients with intra-PT (suspected or confirmed) identified from the overall cohort of HVAD implants from March 2006 to January 2019. The pump thrombus group did not contain data with diagnosed pre-PT or outflow graft occlusion, due to the mechanisms involved resulting in different trends in power consumption [6,8]. A suspected PT was defined by the presence of major hemolysis, heart failure not explained by structural heart disease or abnormal pump parameters together with an accompanying intervention. This intervention or event was either intravenous treatment (anticoagulation, thrombolytics or anti-platelet therapy), pump replacement/exchange/deactivation, urgent transplant listing, stroke, transient ischemic attack or death. Major hemolysis is defined as elevated lactate dehydrogenase levels (LDH) > 2.5 times the upper limit or plasma-free hemoglobin levels > 20 mg/dL with hemoglobinuria, anemia, hyperbilirubinemia or abnormal pump parameters [2].

The time period for the HVAD logfile data snapshots of the PT group was pre-defined with up to 14 days prior to the diagnosed PT until 1 day afterwards. PT event dates and times were recorded based on diagnosis. Data of multiple PT events in the same patient were only used if the time between events was greater than 14 days.

The control group consisted of patients implanted with an HVAD between January 2012 and March 2018 with no history of intra-PT. Pump logfile data of the control group were only used for periods with normal blood levels, no (re-)admission and no hypertension (mean arterial pressure < 110 mmHg). Normal blood levels were defined as c-reactive protein (CRP) < 3 mg/dL, leukocytes < 10 G/L, hemoglobin > 8.5 g/dL and free hemoglobin < 20 mg/dL. Consecutive pump logfile data of the control group with durations < 14 days were excluded from analysis.

### 2.3. Data Analysis

#### 2.3.1. PT Tracking Algorithm

A power-tracking algorithm to detect abnormal pump power was applied on the patient’s pump logfile data. The algorithm is a combination of four independent detectors of abnormal power, as described by Slaughter et al. [13]. The first detector identifies transient variations in the HVAD power waveform by comparing two timescales: a long-term moving average estimates the patient’s historical baseline power consumption, and a short-term moving average indicates transient deviations in the patient’s current operating power from the baseline. The integrated difference between the outputs of the short-term and long-term moving averages provides a quantitative measure of patient-specific power change that may be associated with evolving thrombus. The second detector continuously monitors instantaneous HVAD power, and an alarm is triggered if the instantaneous power exceeds a predefined percentage of the long-term moving average. The third detector triggers an alarm if the instantaneous power exceeds a predefined percentage of the HVAD expected power for the current set speed. The fourth detector is similar to the third, but only operates during the first 24 h immediately after initialization (e.g., after pump start or speed change). The thresholds employed by the algorithm were previously trained with data unrelated to the current study and optimized to minimize false positives while maintaining sensitivity [13]. In addition to the pump power, the power-tracking algorithm occasionally uses pump impeller speed as the second input to readapt an individual’s baseline level following manual adjustments in pump operating speed by the clinical team.

#### 2.3.2. Constant Power Algorithm

In accordance with the typical clinical interpretation of pump performance data, pump power values were compared to constant threshold levels. Pump power threshold levels were added to the baseline power, calculated as the average of the first 5 days of pump logfile data, and compared to the subsequent data in order to detect high power abnormalities. The threshold on the differences in pump power was modulated from 0.1 to 2.5 W with 0.1 W increments to produce a receiver operating characteristics (ROC) curve.

#### 2.3.3. Retrospective Annotation of PT Snapshots

In addition to clinical PT detection performance, PT cases were manually sub-categorized to report on detection performance for different manifestations of PT. PT snapshots were annotated binary by VAD experts in terms of visually observable abnormal pump power indicative for PT (yes/no). The annotation “yes” equals the level of certainty in interpreting pump power signatures leading up to the clinical decision to perform blood laboratory testing. Additionally, PT snapshots were annotated trinary by classifying pump power signatures as indicative of PT as certain, uncertain or absent of abnormalities.

### 2.4. Statistical Analysis

Statistical analysis was performed using MATLAB R2020a (The MathWorks Inc., Natick, MA, USA). The Shapiro–Wilk test was used for numerical data to determine if the null hypothesis of composite normality is a reasonable assumption. The means (±standard deviation) were calculated for normal distributed numerical data and statistical comparisons between two independent continuous variables were performed with Student’s *t*-test. The medians (25th to 75th percentiles, quartile 1 to 3) were calculated for non-normal distribution and statistical comparisons were performed with the Mann–Whitney U-test. Ordinal and categorical data were presented as frequency with percentages, and Pearson’s chi-square test was used to assess statistical significance. Subgroup comparisons according to the three annotated labels of observable power abnormality were performed with the analysis of variance (ANOVA). Statistical significance was set at *p* < 0.05.

A two-term exponential model of LDH levels for the PT group (Appendix A) was calculated and compared with 95% confidence intervals for the control group.

Circadian rhythm was analyzed in terms of seasonality; the frequency at the maximum power spectrum was calculated by Fast Fourier Transform matched 24 h ± 30 min [17].

For the conversion of binary power-tracking outputs for each data sample into overall alarm performance, the following definitions were applied. Sensitivity was calculated from the clinically diagnosed PT group by the number of snapshots with abnormal classified pump data points prior to PT diagnosis divided by the total number of PT snapshots. The specificity of the algorithm was calculated for the control group by the number of pump data logs without abnormal power detection divided by the total number of pump data logs in the control group. Abnormal power detections in the PT group prior to clinical confirmation of PT were classified and reported as early warnings (duration between the first occurrence of the alarm and the PT event date). The calculation of false detections per patient year considers the overall duration of pump log data in the control group divided by the number of pump data logs with abnormal power detections (false positive).

## 3. Results

**Figure 1:**
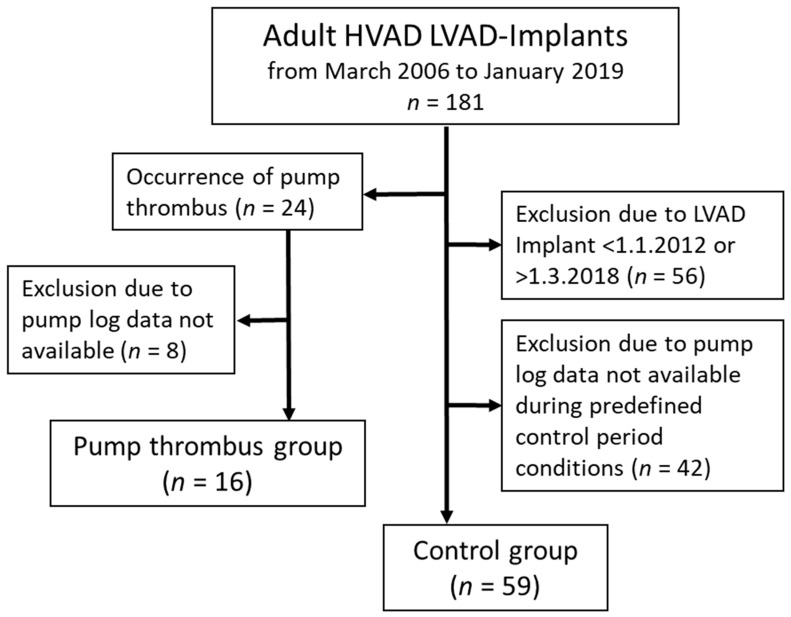
Patient inclusion and exclusion overview for the PT group and control group.

In total, *n* = 75 patients were analyzed, with *n* = 59 patients in the control group and *n* = 16 patients in the PT group (see Figure 1). Median age of the patients was 58.0 (52.0–65.8) years, body mass index (BMI) was 26.2 (23.3–30.3) kg/m² and 18.7% were female. Baseline demographics and comorbidities stratified by PT and control groups are summarized in Table 1. In the PT group, *n* = 30 snapshots from *n* = 16 patients containing *n* = 15 days of pump logfile data were analyzed. In the control group, the duration of logfile data was 98.0 (42.5 to 226.8) days per patient with *n* = 4 (2 to 6) pump log periods with a median duration of 43.5 (31.6 to 53.1) days per patient. The total analyzed pump logfile data duration was 35.9 patient years, with 1.2 patient years for the PT group (3.4%) and 34.7 patient years for the control group (96.6%).

### 3.1. Algorithm and Constant Threshold Performance: Sensitivity, Specificity and Accuracy

Abnormal power detection performance for the power-tracking algorithm and comparison with the best performing constant power threshold (1 W) and the current standard-of-care threshold of 2 W are shown in Table 2. The ROC analysis for different constant power thresholds can be found in Appendix B. PT events were diagnosed by abnormal blood laboratory values with observable pump power signatures in 19 of 30 snapshots. The sensitivity of the power-tracking algorithm was 94.7% for PT snapshots with observable abnormal power signatures indicative for PT and 63.6% for the PT snapshots without abnormal power signatures, (*p =* 0.03). PT snapshots annotated by VAD experts with “observable” (*n =* 17), “uncertain” (*n =* 9) and “absent” (*n =* 4) abnormal power resulted in sensitivities of 88.2%, 77.8% and 75.0%, respectively (*p =* 0.7).

### 3.2. Early Warnings for PT

**Figure 2:**
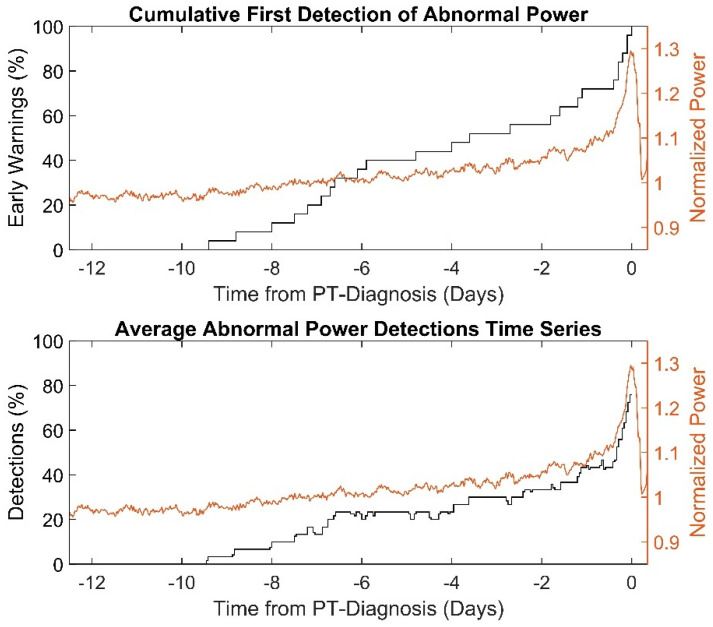
First detection of abnormal pump power cumulated across PT snapshots (top) and abnormal detections time series in the PT snapshots (bottom) with average normalized pump power signature (right ordinates).

The first abnormal power detections of the power-tracking algorithm in PT snapshots, so-called “early-warnings”, occurred 3.6 (6.8 to 0.4) days prior to PT diagnosis. Cumulative first occurrence of abnormal power detections and time series averages in abnormal pump power detections as percentages are shown in Figure 2. The first abnormal power detections of PT snapshots with and without observable pump power abnormalities occurred −1.7 days (−5.9 to −0.3) vs. −5.9 days (±3.1) prior to diagnosis by laboratory parameters (*p =* 0.04). The PT snapshots annotated with abnormal power “observable”, “uncertain” and “absent” showed first abnormal occurrences at −1.6 (−5.6 to 0.3), −4.9 (±3.0) and −5.6 (±5.0) days prior to PT diagnosis (*p =* 0.2), respectively.

### 3.3. False Positives

For the power-tracking algorithm, one pump logfile period with false positive detection was observed every 11.6 patient years. The durations of abnormal power detection in the three false positive logfile periods were 0.3, 1.8 and 7.8 days. For the constant threshold algorithm, the 1 W threshold from a baseline calculated over 1 and 5 days resulted in false positive detections every 2.2 and 2.7 patient years. For the 2 W threshold, false positive detections every 34.7 patient years were observed for both baselines of 1 and 5 days.

### 3.4. Circadian Variation

In the PT snapshots (*n =* 30), a circadian rhythm of LVAD power was observed in 63.3% (*n =* 19). Power-tracking algorithm performance with and without circadian rhythm was 78.9% vs. 90.9% (*p =* 0.4). Furthermore, abnormal power detections within true positive PT snapshots were numerically lower with and without circadian variation (*n =* 19 vs. 11 snapshots), with *n =* 122 (28 to 324) data points vs. *n =* 399 (40 to 489) data points (*p =* 0.2). The first occurrence of true positive detection was significantly earlier without circadian variation at −6.5 (−8.0 to −4.8) days compared to −1.6 (−3.9 to −0.4) days for PT snapshots with circadian variation (*p =* 0.04).

### 3.5. Abnormal Observable Power—Expert Annotation

**Figure 3:**
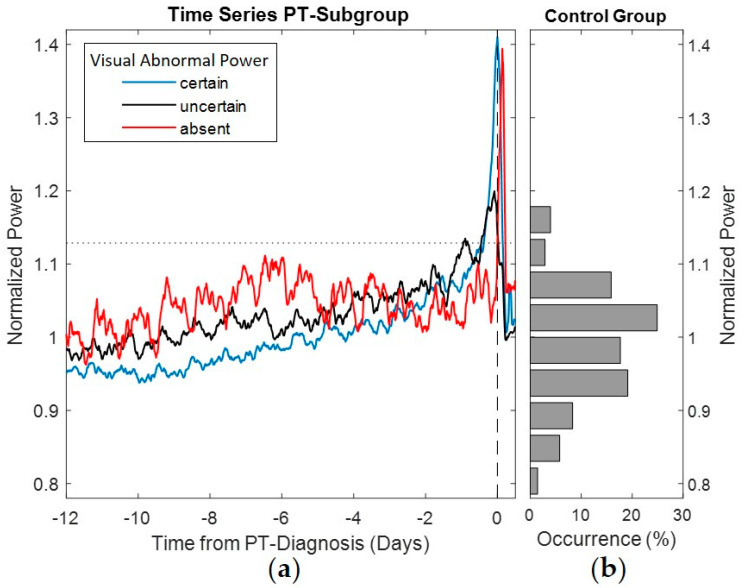
(**a**) Normalized power signature averages for PT snapshot subgroups based on expert annotation (control group’s normalized power 95th percentile, horizontal dotted line; time of PT diagnosis, vertical dashed line) and (**b**) histogram of the normalized power in the control group.

The pump power normalized by expected power corresponding to pump speed was used to calculate the average power signatures of the PTs and to perform a comparison with the power distribution in the control group (see Figure 3). The average power signatures were calculated for each of the annotated observations of abnormal power before PT detection. The gradual build-up in pump power was more prominent in snapshots annotated with “observable” abnormal pump power than for “uncertain” power trends. Snapshots annotated with “absent” abnormal pump power showed abstruse variation and were not indicative of PT by the power signature prior to clinical diagnosis. Nevertheless, a sudden build-up after PT diagnosis occurred. Descriptive statistics of the PT snapshots annotated by abnormal power and detection performance are provided in Table 3.

### 3.6. Parameters Associated with PT

Clinical parameters typically observed during medical check-ups, pump speed and surgical implant access approach were compared for the PT and control groups (see Table 4). Among the statistically different parameters are mean arterial pressure, leukocytes and CRP.

### 3.7. LDH Exponential Model

**Figure 4:**
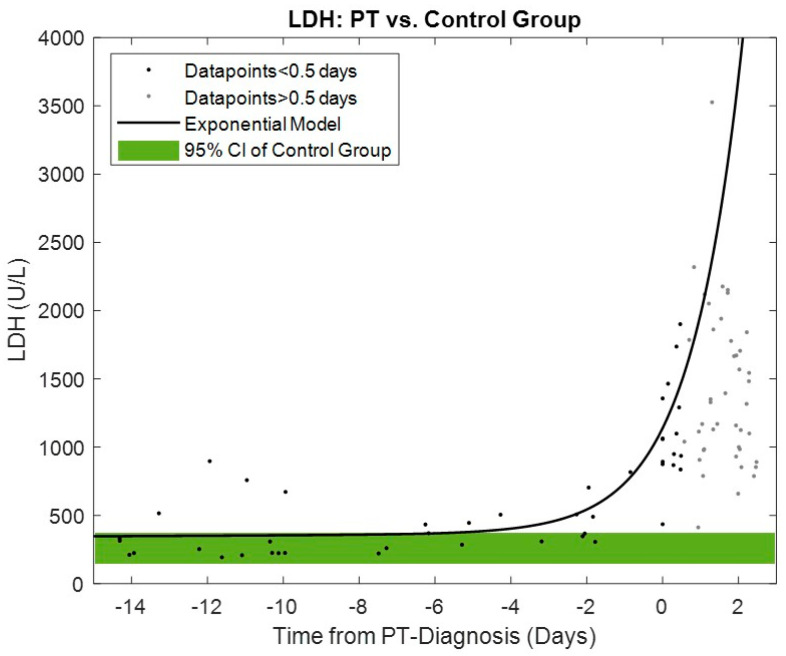
Exponential model for LDH for PT group data points < 0.5 days and 95% confidence interval of control group.

The time series trend of LDH for the PT group compared to the 95% confidence interval ranges in the control group is shown in Figure 4. A significant increase in the LDH exponential model begins within four days prior to PT diagnosis.

## 4. Discussion

In this work, a pump power-tracking algorithm [13] applied to real-world patient logfiles of a centrifugal LVAD was validated, and the results show promising implications.

The algorithm, designed for a low number of false alarms, resulted in high specificity with 98.9%, a crucial value for clinical acceptance of an alarm system. In this study, false alarms occurred every 11.6 patient years, a satisfactory value to prevent false alarm complacency, as mentioned by Hohmann et al. [18]. The benefit of the algorithm was evident with the detection of abnormal power in 83.3% of PT cases and early warnings with a median of 3.6 days prior to PT diagnosis, which could provide meaningful data for early clinical intervention without the need for pump exchange. Furthermore, this study showed novel variability in pump power signatures and reports algorithm performance measures based on circadian variation as well as the comparison to a clinical available optimized alerting system.

The power-tracking algorithm performance was similar to the clinical performance analysis reported in Slaughter et al. [13] with a sensitivity of 85.7% and average early warnings occurring 3.9 days before clinical presentation. However, a more detailed documentation of PT diagnosis and sub-analyses of power trends are presented in this study. The retrospective PT power signatures depend on the timing of diagnosis. Therefore, in some cases, abnormal blood laboratory values may be detected during routine clinical practice without evidence of abnormal observable pump power. In other cases, blood tests are performed after the patient, a caregiver or the VAD clinicians recognize abnormal pump power or symptoms such as hematuria. Depending on the degree of increase in pump power, the standard of care alarm that alerts after an +2 W power increase may be alerting [10]. To better differentiate between these situations, PT snapshots were annotated on the basis of relevance if the trend in pump power was indicative of PT to perform further blood laboratory testing. Indeed, of the *n =* 30 PT snapshots, *n =* 11 showed no trends indicative for PT prior to diagnosis, but there was an increase in power consumption after diagnosis based on laboratory findings. The use of an auto-adaptive system such as the power-tracking algorithm showed the capability to detect abnormalities in the snapshots diagnosed without visual pump power abnormalities, with a sensitivity of 63.6% and early warnings with an average of −5.9 days. This demonstrates the far superior performance of the power-tracking algorithm in detecting abnormalities in these PT cases where no clear power build-up was evident from VAD expert annotation. Permanent monitoring of the pump power with the algorithm integrated into the controller could have tremendous clinical value, as it has been shown that medical therapy is more likely to be successful if started immediately after the first signs of intra-PT [7].

Interpretation of the pump data signature as an early indicator of PT becomes even more challenging in the presence of “signal noise” that may result from hemodynamic instability, suction events [19] and circadian variation [11]. Indeed, PT snapshots annotated with observable pump power abnormalities had a lower percentage of circadian rhythm compared to PT snapshots annotated with uncertain and absent abnormalities. These differences in circadian variation can be observed from the pump power time series averages in Figure 3. Furthermore, all performance measures in the PT group such as sensitivity, early warning and abnormal power detections resulted in better values without circadian variation compared to snapshots with circadian variation.

In addition to the general individual variations in normal pump power, the build-up in pump power due to PT may also vary. Jorde et al. [6] reported the different increases in pump power associated with PT as sudden and gradual build-ups. In the current study, sudden build-ups before PT diagnosis were additionally analyzed by differences in pump power greater than 0.75 W. PT snapshots with observable abnormal pump power showed sudden build-ups in 52.9%, compared to 11.1% and 0% with uncertain and absent abnormal pump power snapshots, respectively. However, as depicted in Figure 3, for PT snapshots absent of abnormal pump power prior to PT diagnosis by blood laboratory values, sudden build-ups in pump power occurred after diagnosis.

The increase in LDH levels associated with PT was exponential, as shown in Figure 4, and began within four days prior to diagnosis, which is also within the mean early warning range of the power-tracking algorithm. Excessive LDH rise or values greater than 1000 U/L occur only very close before diagnosis. Normal LDH levels, indicating no evidence of hemolysis, may be present during the onset of the early warning period, as shown in this study and reported by other colleagues [10,20]. This could lead to a more rigorous and frequent monitoring of such a patient and possibly to risk-adjusted changes in medical therapy. Nevertheless, previously reported risk factors for intra-PT such as elevated MAP [4], infection [21], younger age and higher BMI [22] have been confirmed in this study. The similar INR values between the PT and the control group point towards similar anticoagulation quality and may rule out patient- and management-related device malfunction.

In this work, the simple available threshold comparison to a predefined power level was also evaluated. Even though a threshold level of +2 W was advised by the pump manufacturer, the constant power analysis showed the best results with a threshold level of +1 W. The superior performance in PT detection by the power-tracking algorithm compared to optimized constant thresholds demonstrates the need as well as the benefit of smarter alarms for current LVAD systems. Recently, HVAD patients were found to have a higher incidence of pump thrombosis than HeartMate 3 (Abbott Inc., Chicago, IL, USA) patients [23]. This was one of the reasons why the HVAD was recently withdrawn from the market by the manufacturer. However, there are still thousands of HVAD-supported patients worldwide who could benefit significantly from the implementation of the power-tracking algorithm. Possibly, such implementation by the manufacturer is unlikely. Based on the results of this study, a more stringent alert threshold of +1 W above baseline for HVAD patients to allow early detection and successful treatment of PT can be beneficial.

This work contains the validation of a power-tracking algorithm with data from real-world patients diagnosed with PT and an event-free control group. The key finding of this study is that the use of the power-tracking algorithm can improve therapy of patients implanted with a centrifugal LVAD by detection and early warnings of possible PT events. A major clinical implication is that abnormal pump power was even classified by the algorithm in snapshots visually lacking pump power abnormalities indicative of PT with superior performance to the constant threshold-based clinical alternative. Patient management tailored to the individual to avoid the risk factors associated with PT, combined with an alerting system using the power-tracking algorithm, could help to mitigate or even prevent the associated adverse effects, and ultimately improve LVAD patient outcomes.

### Limitations

This single-center study with limited patient numbers does not provide sufficient statistical power to generalize results to other centers without further clinical evidence. The retrospective study design with a long observation period could potentially include variations in patient management strategies. The control cohort was defined using multiple parameters, which was necessary to conduct this validation study. There were unbalanced data between the groups. The relevance of this study has diminished with the HVAD being taken off the market; nevertheless, such an algorithm could be useful for any centrifugal LVAD, such as the HeartMate 3, and potentially also for axial LVADs, such as the Impella (Abiomed Inc., Danvers, MA, USA).

## 5. Conclusions

To conclude, in this study, a pump power-tracking algorithm applied to real-world LVAD patient logfiles was validated, and the results show promising implications. The algorithm detected intra-PT with high sensitivity (83.3%) and a low false positive rate (every 11.6 patient years), and provided early warnings with a median of 3.6 days prior to PT diagnosis, which could provide meaningful guidance for early clinical intervention without the need for pump exchange.

## Figures and Tables

**Table 1:** Patient demographics and cardiac parameters at implant.

	All	Control Group	PT Group	*p*-Value
Patients	*n* = 75	*n* = 59	*n* = 16	
Gender	Male: *n* = 61 (81.3%)	Male: *n* = 49 (83.1%)	Male: *n* = 12 (75.0%)	*p =* 0.5
	Female: *n* = 14 (18.7%)	Female: *n =* 10 (16.9%)	Female: *n =* 4 (25.0%)	
Age (yrs)	58.0 (52.0–65.8)	60.0 (53.0–66.0)	54.0 (43.5–60.0)	*p =* 0.07
Weight (kg)	78.0 (72.3–91.0)	76.0 (70.0–90.0)	86.5 (76.5–96.5)	*p =* 0.05
Height (cm)	175 (±10)	175 (±9)	174 (±10)	*p =* 0.8
BSA (m²)	2.0 (1.8–2.1)	1.9 (1.8–2.1)	2.1 (1.9–2.1)	*p =* 0.1
BMI (kg/m²)	26.2 (23.3–30.3)	25.4 (23.0–29.6)	29.1 (25.0–31.1)	*p =* 0.02
Blood type	0 neg: *n* = 2 (2.7%)	0 neg: *n* = 2 (3.4%)	0 neg: *n* = 0 (0.0%)	*p =* 0.6
	0 pos: *n* = 14 (18.7%)	0 pos: *n* = 12 (20.3%)	0 pos: *n* = 2 (12.5%)	
	A neg: *n* = 7 (9.3%)	A neg: *n* = 6 (10.2%)	A neg: *n* = 1 (6.3%)	
	A pos: *n* = 29 (38.7%)	A pos: *n* = 20 (33.9%)	A pos: *n* = 9 (56.3%)	
	AB pos: *n* = 5 (6.7%)	AB pos: *n* = 5 (8.5%)	AB pos: *n* = 0 (0.0%)	
	B neg: *n* = 3 (4.0%)	B neg: *n* = 2 (3.4%)	B neg: *n* = 1 (6.3%)	
	B pos: *n* = 15 (20.0%)	B pos: *n* = 12 (20.3%)	B pos: *n* = 3 (18.8%)	
Cardiomyopathy	ischemic: *n* = 45 (60.0%)	ischemic: *n* = 35 (59.3%)	ischemic: *n* = 10 (62.5%)	*p =* 0.8
	non-ischemic: *n* = 30 (40.0%)	non-ischemic: *n* = 24 (40.7%)	non-ischemic: *n* = 6 (37.5%)	
Intermacs Level	1: *n* = 21 (28.0%)	1: *n* = 19 (32.2%)	1: *n* = 2 (12.5%)	*p =* 0.04
	2: *n* = 14 (18.7%)	2: *n =* 8 (13.6%)	2: *n =* 6 (37.5%)	
	3: *n =* 25 (33.3%)	3: *n =* 18 (30.5%)	3: *n =* 7 (43.8%)	
	4: *n =* 15 (20.0%)	4: *n =* 14 (23.7%)	4: *n =* 1 (6.3%)	
Outflow graft anastomosis to	A. subcl. Dext.: *n =* 1 (1.3%)	A. subcl. Dext.: *n =* 1 (1.7%)	A. subcl. Dext.: *n =* 0 (0.0%)	*p =* 0.7
	A. subcl. sin.: *n =* 19 (25.3%)	A. subcl. sin.: *n =* 14 (23.7%)	A. subcl. sin.: *n =* 5 (31.3%)	
	Asc. Aorta: *n =* 55 (73.3%)	Asc. Aorta: *n =* 44 (74.6%)	Asc. Aorta: *n =* 11 (68.8%)	
Intraventricular thrombus present during implantation	no: *n =* 74 (98.7%)	no: *n =* 58 (98.3%)	no: *n =* 16 (100.0%)	*p =* 0.6
	yes: *n =* 1 (1.3%)	yes: *n =* 1 (1.7%)	yes: *n =* 0 (0.0%)	
Bilirubin (mg/dL)	1.1 (0.7–2.0)	1.2 (0.7–2.1)	1.0 (0.8–1.7)	*p =* 0.6
Creatinine (mg/dL)	1.3 (1.0–1.7)	1.2 (1.0–1.6)	1.3 (1.1–1.9)	*p =* 0.6
Meld-Score	13.3 (±6.1)	13.4 (±6.0)	13.1 (±6.6)	*p =* 0.9

yrs, years; BSA; body surface area; BMI, body mass index; Intermacs, Interagency Registry for Mechanically Assisted Circulatory Support; Meld, model of end stage liver disease; PT, pump thrombus.

**Table 2:** Performance measures to detect abnormal pump power prior to diagnosed PT for the power-tracking algorithm, 1 Watt and 2 Watt threshold-based algorithms.

	Power-Tracking	Constant Threshold 1W, Baseline 1 Day	Constant Threshold 1W, Baseline 5 Days	Constant Threshold 2W, Baseline 1 and 5 Days *
Sensitivity (%)	83.3%	86.7%	86.7%	36.7%
Specificity (%)	98.9%	94.2%	95.3%	99.6%
Accuracy (%)	96.1%	92.3%	93.2%	93.5%

* Results for constant threshold 2 W with a baseline of 1 and 5 days were identical.

**Table 3:** Descriptive statistics of PT snapshots and power-tracking classification performance stratified by abnormal power VAD expert annotation.

	Abnormal Power Expert Annotation			*p*-Value
	Observable	Uncertain	Absent	
PT snapshots, *n* (%)	17 (57%)	9 (30%)	4 (13%)	*p =* 0.3
Sensitivity,%	88.2%	77.8%	75.0%	*p =* 0.7
Abnormal Power Detections, (n-datapoints)	152 (25–438)	250 (±188)	384 (±359)	*p =* 0.7
Early Warning (days)	−1.6 (−5.6 to −0.3)	−4.9 (±3.0)	−5.6 (±5.0)	*p =* 0.2
Circadian variation, *n* (%)	10 (59%)	6 (67%)	3 (75%)	*p =* 0.8

**Table 4:** Potential risk factor comparison for the PT and control group.

	PT Group	Control Group	PT vs. Control Group
LVAD speed (rpm)	2696 (2636–2796)	2796 (2596–2895)	*p =* 0.5
Mean arterial pressure (mmHg)	85.5 (82.5–94.3)	81.7 (76.5–85.4)	*p* < 0.005
INR	2.3 (2.0–2.7)	2.4 (2.1–2.8)	*p =* 0.2
Circadian variation in LVAD power (% of pats)	63.3%	72.2%	*p =* 0.3
Leukocytes (G/L)	9.2 (7.9–11.4)	7.6 (6.5–9.0)	*p* < 0.005
CRP (mg/dL)	1.4 (1.1–1.7)	0.6 (0.3–1.1)	*p* < 0.0001
Minimal invasive surgical approach (% of pats)	80.0%	69.5%	*p =* 0.5

**Figure A1:**
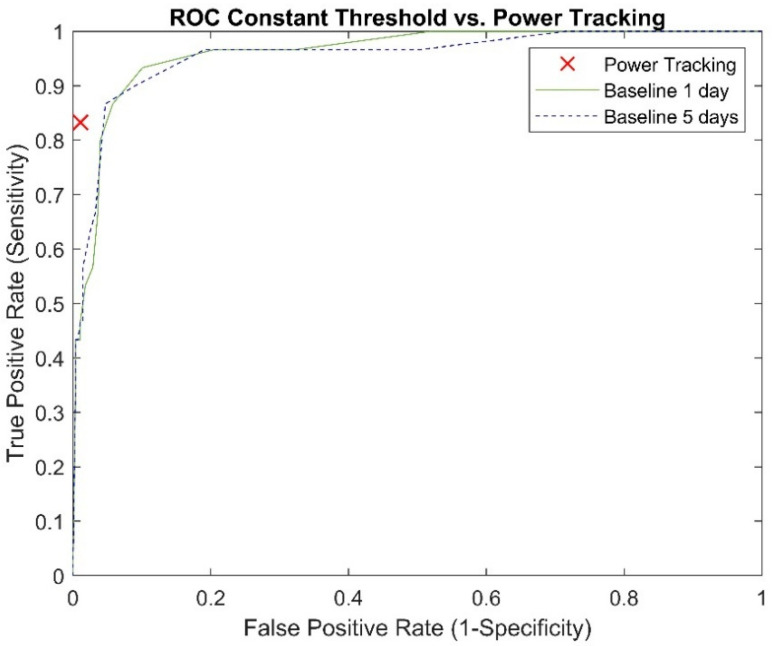
Receiver operating characteristic analysis of the power tracking algorithm, and with constant threshold (from 0.1 to 2.5 W) for a baseline of 1 and 5 days to determine pump power consumption baseline.

## Data Availability

The data are not publicly available due to agreements with the Ethics Committee of the Medical University of Vienna.

## Appendix A

Two-term exponential model formula:

f (x) = a ∗ exp(b ∗ x) + c ∗ exp(d ∗ x)

## Appendix B

ROC analysis was performed for a baseline of 1 and 5 days to determine pump power consumption baseline, with the constant threshold for detection varying from 0.1 to 2.5 W.
